# Savage Nature and Ecologic Exchange

**DOI:** 10.3201/eid1912.AC1912

**Published:** 2013-12

**Authors:** Polyxeni Potter

**Affiliations:** Centers for Disease Control and Prevention, Atlanta, Georgia, USA

**Keywords:** art science connection, emerging infectious diseases, art and medicine, Savage Nature and Ecologic Exchange, Paul Gauguin, one medicine, zoonoses, Black Pigs, about the cover

**Figure:**
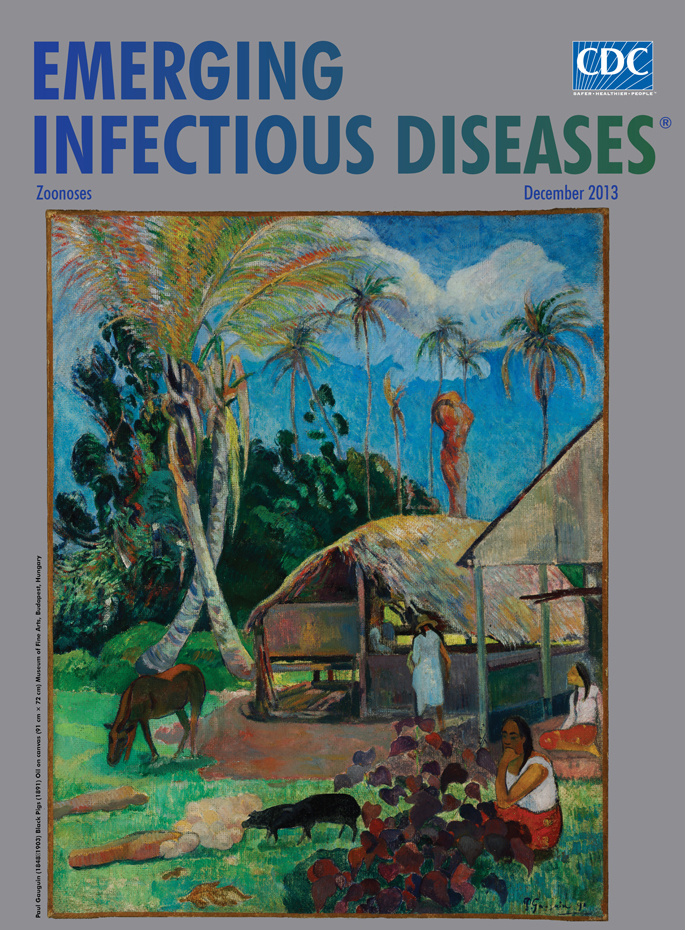
**Paul Gauguin (1848‒1903) *Black Pigs* (1891) Oil on canvas (91 cm × 72 cm)** Museum of Fine Arts, Budapest, Hungary

“There are neither carnivores nor reptiles in Tahiti,” wrote Paul Gauguin in his journal Noa Noa. “The only ‘wild game’ on the island are the pigs which have escaped into the forest, where they have multiplied and become entirely wild.”

The artist’s first impression after the long voyage, “sixty-three days of feverish expectancy,” was “nothing very extraordinary; nothing, for instance, that could be compared with the magnificent bay of Rio de Janeiro.” A native Parisian, Gauguin had spent his childhood in South America, where his family had ties to Spanish nobility in Peru. His mother collected pre-Columbian pottery. These early life experiences he mythologized into a complex persona, often referring to himself as part savage. “You know that I have Indian blood, Inca blood in me, and it’s reflected in everything I do…. I try to confront rotten civilization with something more natural, based on savagery.”

This desire to escape his own times and the vestiges of a civilization he considered corrupt and dehumanizing guided Gauguin’s work. He came to art late in life, first cautiously as a collector; then for pleasure, as friend of Edgar Degas, Paul Cézanne, and Camille Pissarro. He became entirely consumed by art when he worked for a time with Vincent van Gogh. “I am a great artist and I know it.” After the stock market crash in 1882, he left Paris to travel and seek the authentic life he believed would lead him to greatness. “You must remember,” he wrote to his wife, “that I have a dual nature, [that of] the Indian and [that of] the sensitive man.”

He moved to the French coast. “I love Brittany; I find the wild and the primitive here. When my clogs resonate on this granite ground, I hear the muffled and powerful thud that I’m looking for in painting.” Then he settled in Martinique “to live as a savage,” but soon he left France altogether. “There in Tahiti,” he wrote to his wife, “in the silence of the tropical night, I shall be able to listen to the sweet murmuring music of my heart’s movements in loving harmony with the beings around me. Free at last with no money troubles and able to love, sing, and die.” He painted his best work during his first of two trips to Tahiti. He died of syphilis nearly penniless in the Marquesas.

During his travels to the South Sea Islands, Gauguin found that many of his expectations for a life of wildness filled with savages and untamed nature existed only in myths and fables. “The Tahitian soil is becoming completely French.” Unable to find the world he dreamed about, he sought his own. This preferred world he created in his art. From the exotic islands he extracted magical colors he then placed side by side in unconventional combinations and clearly outlined. Despite his admiration for and friendship with the impressionists, he shunned their work in favor of the flat planes that anticipated modern art.

*Black Pigs*, on this month’s cover, was painted the first year Gauguin was in Tahiti. Although he had not yet entirely abandoned perspective, spatial relationships are secondary to color and form. Traces of impressionist technique appear in the brushwork of the foliage and thatched roof of the hut. But the human and animal figures, including the namesake pigs, are simplified, almost archaic in their peacefulness, and entirely at ease with the surroundings.

In island culture, the sacred pig was the bond between gods and humans, facilitating their intermarriages or mitigating their disputes. “Food for gods and men” and the backbone of household prosperity, pigs were mentioned in Gauguin’s writings again and again, his cordial interactions with island neighbors sprinkled with “daintily prepared little pig” or “little pigs roasted on hot stones.”

Tahiti “Is the summit of a mountain submerged at the time of one of the ancient deluges,” the artist wrote. “Only the very point rose above the waters. A family fled thither and founded a new race—and then the corals climbed up and along it, surrounding the peak, and in the course of centuries builded a new land. It is still extending, but retains its original character of solitude and isolation, which is only accentuated by the immense expanse of the ocean.” Yet even this isolation that Gauguin sensed so acutely was an illusion. During a period of exotic introductions, local animals were modified along with the political, economic, spiritual, social, and physical landscape.

Animal movement and trade around the globe, which already had altered the island fauna in Gauguin’s diminishing paradise, now have eliminated even the possibility of zoonotic isolation. As animals and the viruses, bacteria, and internal and external parasites that travel with them find themselves evolving independently from their forbearers in new ecologic niches with new pressures and pathogenic tools, genetic changes occur in them. These changes facilitate emergence of new pathogens that can then start the process over again as the pathogens continue to span the globe.

Island people, Gauguin believed, “Had been richly endowed with an instinctive feeling for the harmony necessary between human creations and the animal and plant life which formed the setting and decoration of their existence.” This harmony, which included the savage element he sought and captured in garish tones, he viewed as a main ingredient of great art, along with the unity of humans with nature. This oneness in nature, with its inherent intimacy of all the macroscopic and microscopic players, requires concurrent attention to human health, animal health, and environmental health. For in this cauldron, as full participants and hosts of the wild microbes, which inhabit and kill us, we remain, like Gauguin, part savages.
